# Inadvertent Placement of Thoracic Epidural Catheter in Pleural Cavity: A Case Report and Review of Published Literature

**DOI:** 10.7759/cureus.37642

**Published:** 2023-04-16

**Authors:** Sanaa Khan, Wajahat Nazir Ahmed, Asad Aleem, Saad Ur Rehman

**Affiliations:** 1 Anesthesia, Shaukat Khanum Memorial Cancer Hospital and Research Centre, Lahore, PAK

**Keywords:** inadvertent, accidental, cavity, pleural, misplaced, catheter, epidural

## Abstract

Thoracic epidural placement is considered the gold standard for pain management for abdominal or thoracic surgery. It provides analgesia superior to that provided by opioids with a decreased risk of pulmonary complications. Insertion of a thoracic epidural catheter requires the knowledge and expertise of an anesthetist; epidural catheter insertion may be challenging especially when sited in the higher thoracic region, in patients with unusual neuraxial anatomy, patients unable to position adequately for insertion or morbidly obese patients.^ ^Postoperatively the anesthetic team is required to look after the patient and assess for any complications such as hypotension. Even though the incidence of complications may be low; however, some of these could have detrimental consequences for the patients such as epidural abscess, hematoma formation, and temporary or permanent neurological damage. In this case report, we will discuss a patient who underwent a three-stage esophagectomy for esophageal squamous cell carcinoma under general anesthesia with epidural analgesia. The epidural catheter (Portex® Epidural Minipack System with NRFit® connector, ICUmedical, USA) was found in the intrapleural space during video-assisted thoracoscopy for the thoracic part of esophagectomy. To facilitate surgical access, the catheter was removed immediately, and the patient was given patient-controlled analgesia with morphine for postoperative pain control.

## Introduction

A thoracic epidural is widely accepted as an excellent mode of analgesia for an array of surgeries including thoracic, upper abdominal, and obstetric surgeries [1]. The epidural can be used to provide pain relief intraoperatively and postoperatively. Thoracic epidural catheter placement requires the knowledge and expertise of an anesthetist; challenges may be faced when sitting in the higher thoracic region, in patients with aberrant neuraxial anatomy, inadequate patient positioning, or morbidly obese patients [2]. Owing to the blind nature of the procedure and certain technical difficulties that may be encountered during the procedure the epidural insertion can result in complications such as postdural puncture headache, nerve trauma, hematoma and abscess formation, and intravascular injection [3]. Misplacement of the epidural catheter into the intrapleural space is rare but not unheard of [4-7]. There are cases in the literature that report a misplaced epidural catheter into the pleural space and utilized it to provide analgesia [8].

This case report highlights another incident of a misplaced epidural catheter in the pleural cavity. A thorough literature review provides a comprehensive outline of the magnitude of a problem that exists in the form of fragmented and isolated case reports or series.

## Case presentation

A 48-year-old gentleman, a known case of biopsy-proven poorly differentiated squamous cell carcinoma esophagus was scheduled for a three-stage esophagectomy. He had no co-morbidities, and his function status was above four metabolic equivalents. A thorough preoperative anesthesia assessment was done and all laboratory investigations were found to be within normal limits. Physical examination and airway were found to be unremarkable. A comprehensive anesthetic plan for three-stage esophagectomy with video-assisted thoracic surgery (VATS) was drawn up, which included general anesthesia and epidural catheter insertion for analgesia. The procedure was explained to the patient in detail and informed consent was taken.

On the morning of the surgery, the patient was shifted to the operating room where his vitals were recorded which were found to be within normal physiological limits. The recorded weight was 51 kilograms and height 160 centimeters with a calculated body mass index (BMI) of 20kg/m^2^. The patient was transferred to the operating room and monitored as per American Society of Anesthesiologists (ASA) guidelines. Epidural catheter insertion was attempted in the midline approach with the patient in a sitting position. The epidural catheter set was opened, and a 16-gauge (G) Tuohy needle was used in the T5-6 interspace. On encountering difficulty in the midline, after two attempts the right-sided paramedian approach was used. The epidural space was found at 7.5cm with a loss of resistance (LOR) to saline while using the continuous pressure method. The catheter was threaded without resistance to 13cm, secured with a dressing, and flushed with lignocaine to rule out intrathecal placement. A “falling meniscus” in the epidural catheter was taken as an additional sign of correct placement.

**Figure 1:**
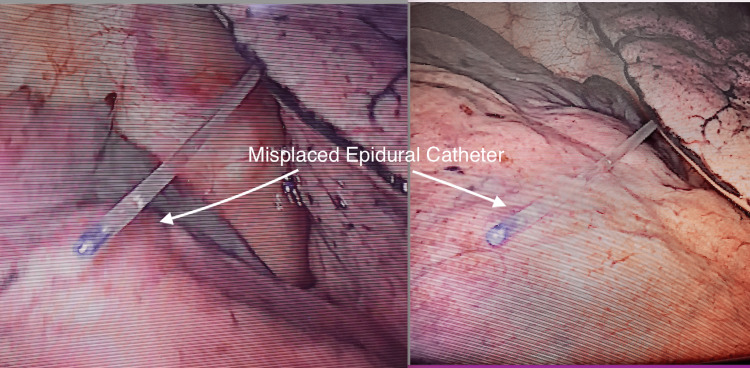
Misplaced intrapleural catheter as seen during video-assisted thoracic surgery (VATS)

Anesthetic induction was done using standard hospital protocols using midazolam, propofol, and atracurium. Intubation was done with a left-sided double-lumen tube 37 French (Fr) and tube position was confirmed by fiberoptic bronchoscopy, and the patient was positioned in the left lateral position for VATS. Prior to the skin incision, the epidural was loaded with 10mL of bupivacaine 0.25% (25mg) and blunting of response to the surgical stimulus was seen. After the surgeon inserted the camera through one of the port sites, the epidural catheter was seen in the right pleural cavity as shown in Figure 1. There was no evidence of intra-thoracic hemorrhage or lung injury. After discussion with the surgical team, the epidural catheter was removed as it was hampering surgical access.

**Figure 2:**
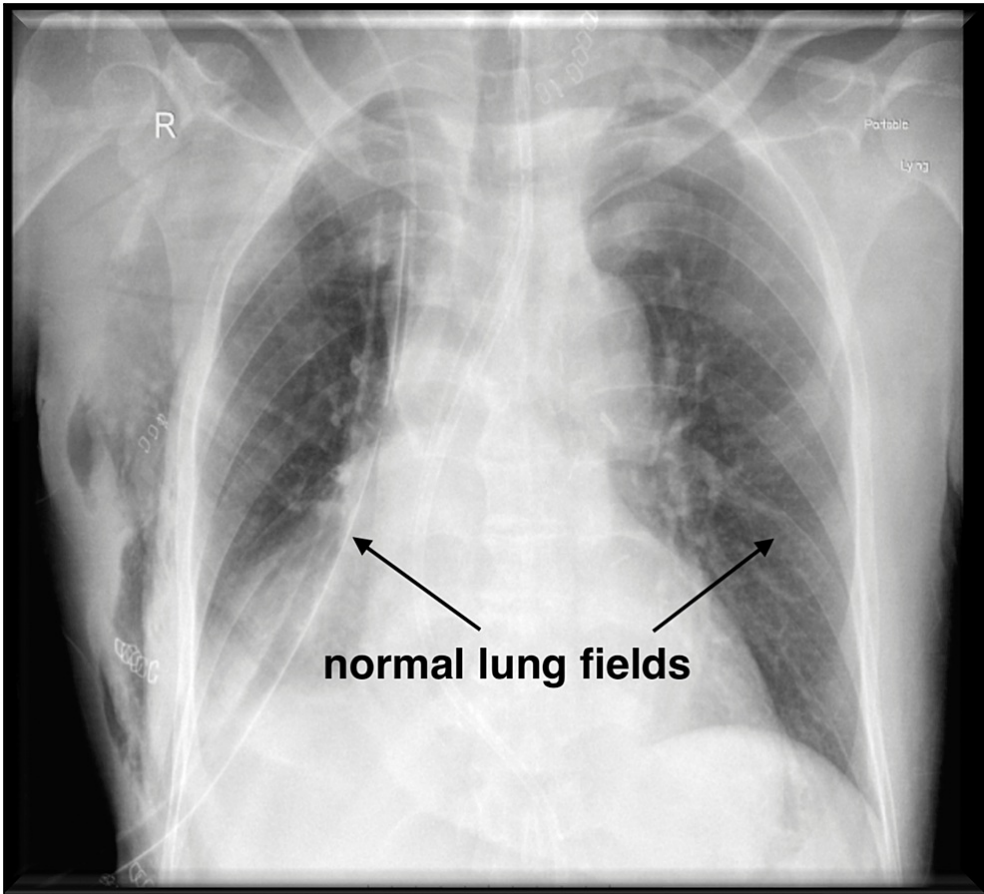
Normal chest x-ray after extubation

Intravenous (IV) morphine and paracetamol were given to the patient for analgesia and rest of the course of surgery remained uneventful. At the end of the surgery, another attempt to place epidural catheter in left lateral position was made by a second operator in the T5-6 interspace using midline approach with a 16G Tuohy needle. LOR was met at 7.5cm and catheter was threaded till 12cm. The patient was given an antiemetic and neuromuscular blockade was reversed using neostigmine and glycopyrrolate. In the immediate postoperative period, a portable chest x-ray was done, and an arterial blood sample was sent for analysis. No acute changes were seen on the chest x-ray as shown in Figure 2, and arterial blood gases were within acceptable limits.

The patient was stabilized and transferred to Post Anesthesia Care Unit (PACU) where his oxygen requirement came down. The epidural was removed since it was found to be ineffective in assessing sensory levels and asking for pain control as per Numerical Rating Scale (NRS). The patient was shifted to IV patient-controlled analgesia as the pain regimen thereafter.

After a smooth course in PACU, the patient was transferred to High Dependency Unit (HDU) for further management. He was nursed in the Intensive Care Unit (ICU) for the next few days due to postoperative pneumonia and lung collapse. He was managed with supplemental oxygen, antibiotics, incentive spirometry, and chest physiotherapy. The pain remained controlled with patient-controlled analgesia using IV morphine with a documented mean pain score of 1.25/10 on NRS. On postoperative day 7, the patient was transferred to inpatient facility and on postoperative day 10, the patient was discharged to home.

## Discussion

Failure of thoracic epidural analgesia may occur in up to 30% of cases, either due to primary misplacement of the catheter or secondarily due to catheter migration due to position change or oscillations within the cerebrospinal fluid (CSF) [2]. To date, LOR with saline remains the most widely used method to localize the epidural space with other methods for localization failing to demonstrate sufficient accuracy or predictability to be incorporated into routine clinical practice [9]. Even with this high rate of failure, intrapleural misplacement of an epidural catheter is considered to be a rare complication that has consistently been reported in the literature in the form of case reports [4-6]. It is possible that accidental misplacement of epidural catheters in the pleural space is an under-reported complication; in our case, it was recognized because the catheter had been misplaced into the ipsilateral pleural cavity where it was seen during VATS whereas it could have gone unnoticed had the catheter been displaced toward the contralateral pleural space. It is pertinent to mention that most of the literature reviewed reported cases in which epidural catheters were found in the pleural cavity during VATS (four cases) or thoracotomy (13 cases). There is a need to be mindful of this complication in view of a failure to achieve adequate epidural analgesia and investigate appropriately. Another significant challenge is a lack of cost-effective investigations that could be used to confirm such a diagnosis. Simple tests, such as aspirating for blood, a test dose with lignocaine and adrenaline, and assessment of sensory levels after awakening epidural catheter placement before induction of anesthesia have been proposed in the literature previously. There have been other methods proposed to correctly identify the intrapleural placement of epidural catheters including routine chest x-rays with a digital enhancement of images making it easier to radiologically identify the catheter [10]. Fluoroscopy guidance for identifying the epidural space has found routine use in chronic pain procedures; however, using fluoroscopy in routine intraoperative care to identify a rare complication would need justification of radiation exposure to patients. Radiological investigations however have found a role in the preoperative assessment of spinal anatomy, computed tomography (CT) scans have been used to identify the depth of the epidural space, which can then be used during the procedure to guide needle advancement and localization of the epidural space [11]. Similarly, ultrasonography can be used during the procedure to guide the epidural needle [12]. Through the literature review, it has been noted that this complication occurs more in cases where the paramedian approach has been used [4]. A possible explanation of this might be the inability to feel a characteristic LOR as ligamentum flavum is not encountered with this approach. A suboptimal LOR and an insertion point that is lateral to the midline may increase chances of, and subsequently, the inability to recognize misplacement of the epidural catheter. Quality of the patient’s anatomical landmarks and adequacy of patient positioning is also identified as independent predictors of successful epidural catheter placement [13] and any difficulty faced with these two factors may lead to an unsuccessful or misplaced epidural catheter.

In some cases, the misplaced catheter has been successfully used to provide analgesia with good results; however, the efficacy of such a technique is questionable. Administration of local anesthetics through the misplaced catheters was proposed as a reasonable analgesic option [8,14]. The consequences of this complication may vary from asymptomatic to potentially life-threatening complications such as pneumothorax or hemothorax [15,16]. Therefore, it is pertinent to anticipate any potential difficulty related to the procedure and investigate pre-emptively in patients who may pose a challenge.

A thorough literature search was performed using PubMed with the keywords “epidural catheter,” “pleural,” “accidental,” and “misplaced.” Case reports or case series which reported accidental placement of epidural catheters in the pleural space between the years 1994 and 2023 were selected for review and comparison. A total of 18 articles were selected.

Out of the 20 misplaced catheters, seven were used to provide analgesia of which 57% were successful in providing adequate analgesia. Short- and long-term complications were reviewed and there were only three reported short-term complications with no long-term complications in any of the cases. Table 1 summarizes the consequences of epidural catheters that were misplaced in the pleural cavity as reported in the literature, whereas Table 2 provides an overview of complications and management mentioned in literature.

**Table 1:** Summary of articles reviewed NA = Not available

Name	Number of cases reported	Misplaced catheter used for analgesia	Pain control adequate	Treatment	Immediate complications	Long-term complications	Diagnosis of misplacement	Approach
Grieve et al. [17]	1	Yes	No	Catheter removed	None	None	Thoracotomy	Paramedian
Zaugg et al. [18]	1	No	Not applicable	Catheter removed	None	None	VATS	Paramedian
Rachna et al. [5]	1	No	Not applicable	Catheter removed	None	None	Thoracotomy	Midline
Furuya et al. [4]	1	No	Not applicable	Catheter removed	None	None	Thoracotomy	Paramedian
Patermann et al. [19]	1	No	Not applicable	Catheter removed	None	None	Thoracotomy	Midline
Sundary et al. [20]	1	Yes	Yes	Not needed	None	None	Thoracotomy	Paramedian
Lin et al. [6]	1	No	Not applicable	Catheter removed	None	None	Thoracotomy	Paramedian
Belani et al. [21]	1	No	Not applicable	Catheter removed	Pneumothorax	None	Thoracotomy	Midline
Inoue et al [8]	3	Yes	Yes = 1 No = 2	Not needed	None	None	1=VATS 2=Thoracotomy	Paramedian
Lida et al. [16]	1	No	Not applicable	Catheter removed	Hemothorax	None	Clinically	Paramedian
Eti et al. [22]	1	No	Not applicable	Catheter removed	None	None	Thoracotomy	Midline
Cordone et al. [23]	1	Yes	Yes	Chest tube insertion	Pneumothorax	None	CT Scan	Paramedian
Li [24]	1	No	Not applicable	Catheter removed	None	None	VATS	Paramedian
Kim et al. [25]	1	No	Not applicable	Catheter removed	None	None	Thoracotomy	Paramedian
Pavithran et al. [26]	1	No	Not applicable	Catheter removed	None	None	Thoracotomy	Paramedian
Alagöz et al. [27]	1	No	Not applicable	Catheter removed	None	None	Thoracotomy	Midline
Von Hösslin et al. [28]	1	No	Not applicable	Catheter removed	None	None	NA	NA
Wagh et al. [29]	1	Yes	Yes	Not needed	None	None	VATS	Midline

**Table 2:** Overview of the complications and management post misplacement

Total number of cases	Used for analgesia	Analgesia adequate	Serious complications
20	7 (35%)	4 (57%)	3 (15%)

Insertion of an epidural catheter requires thorough knowledge of the neuraxial anatomy. During placement, one should be mindful of the possible complications that may occur. Multiple attempts should be avoided, and senior help must be sought in case difficulty is encountered during insertion. Radiological guidance may be utilized in challenging cases. The anesthetist should not rely solely on LOR, hanging drop method or “meniscus fall” as these can give a false impression of being in the epidural space because all three will occur in case the catheter enters the pleural space as well. It is preferable to insert the epidural catheter while the patient is awake since insertion can be guided and tested for blockade with a test dose of local anesthetic to rule out intrathecal or intravascular placement.

## Conclusions

This case was another incident of inadvertent intrapleural placement of an epidural catheter which was incidentally found during VATS. There were no short- or long-term sequelae. This incident further highlights the fact that such misplacement may not be clinically evident and a high degree of suspicion in case of a “failing epidural,” with or without pre-existing risk factors for difficult placement of the epidural catheter, may be warranted. Being wary of complexities involved in any procedure is crucial as it can make the anesthetist more heedful toward the implications. When a complication occurs, it is important to plan the way forward including how to deal with consequences, alternatives, and how to avoid it in the future. Since there are no practical bedside investigations available, a high index of suspicion is warranted in case of a failing epidural, unilateral or patchy sensory/motor blockade, or respiratory distress secondary to pneumothorax or hemothorax. The presence of any clinical signs mentioned above should prompt the clinician to investigate further with imaging if possible.
